# Immunogenicity and clinical features relating to BNT162b2 messenger RNA COVID-19 vaccine, Ad26.COV2.S and ChAdOx1 adenoviral vector COVID-19 vaccines: a systematic review of non-interventional studies

**DOI:** 10.1186/s43094-022-00409-5

**Published:** 2022-03-26

**Authors:** Chinonyerem O. Iheanacho, Uchenna I. H. Eze

**Affiliations:** 1 Department of Clinical Pharmacy and Public Health, Faculty of Pharmacy, University of Calabar, PMB 1115, Calabar, Nigeria; 2 Department of Clinical Pharmacy and Biopharmarcy, Faculty of Pharmacy, Olabisi Onabanjo University, Sagamu, Nigeria

**Keywords:** COVID-19 vaccines, Messenger RNA vaccines, Adenovirus vector-based vaccines, Immunological effects, Thrombosis, Thrombocytopenia, BNT162b2, ChAdOx1, Ad26.COV2.S

## Abstract

**Background:**

Vaccination against Coronavirus disease 2019 (COVID-19) is an important means of controlling the pandemic, however they are expected to stimulate immune responses when administered to confer immunity. In this review, we evaluated the clinical and laboratory features associated with BNT162b2 messenger RNA COVID-19 vaccine, Ad26.COV2.S and ChAdOx1 adenoviral vector COVID-19 vaccines, to determine their immunogenicity. Demographic distribution of pathogenic autoimmune response and time interval between vaccination and onset of symptoms were also assessed. This was to identify; persons at risk of developing auto-immune reactions and  markers to enhanced occurrence of this event.

**Main body:**

Using relevant keywords, search was conducted in the databases of PubMed, Scopus, Web of Science and Google scholar from November 2020 to May 31, 2021. Additional article was also identified through hand-searching of reference lists, and the review was conducted in line with Preferred Reporting Items for Systematic Reviews and Meta-Analysis (PRISMA) guidelines 2009. Study outcome measures were presence of antibodies after vaccination and evidence of autoimmune reactions, therefore studies relating these measures were considered eligible for this review. Studies showed stimulation of immune response with administration of BNT162b2 mRNA vaccine, ChAdOx1 and Ad26.COV2-S adenovirus vector-based vaccines. Aside SARS-CoV-2 spike protein antibodies, elevated D-dimers, presence of PF4 and low fibrinogen were most commonly seen laboratory features in persons with autoimmune reactions following vaccination. In addition, thrombotic thrombocytopenia was the commonest clinical features observed with ChAdOx1 and Ad26.COV2-S adenovirus vector-based vaccines. Findings from this study also suggest higher susceptibility of women of 22–60 years to the pathogenic immunogenicity that may particular result from exposure to ChAdOx1 and Ad26.COV2-S adenovirus vector-based vaccines. Time interval of 4–37 days was mostly observed between vaccination and occurrence of a symptom.

**Conclusion:**

Immune thrombotic thrombocytopenia and other PF4 dependent syndrome are likely associated with ChAdOx1 and Ad26.COV2.S adenovirus vector vaccines, mostly occurring in women usually within 4–37 days of first dose of vaccine. Enhanced knowledge about vaccine adverse effects and its distribution is crucial for effective vaccination strategies.

## Background

As an ongoing pandemic, coronavirus disease 2019 (COVID-19) has caused significant disruption in social and economic lives of persons [1]. Caused by a new virus (Severe Acute Respiratory Syndrome Coronavirus 2—SARS-CoV-2), the pandemic necessitated the development of various vaccines for its effective control as no drug is currently approved for its cure although some have been associated with improved outcomes [2–4]. Two main types of SARS-CoV-2 vaccines are currently authorised for emergency use—the messenger Ribonucleic Acid vaccines and the adenovirus vector-based vaccines [5]. More so, massive vaccination campaigns which has seen the vaccination of several millions of persons [6] brings hope for the end of the pandemic.

**Fig. 1:**
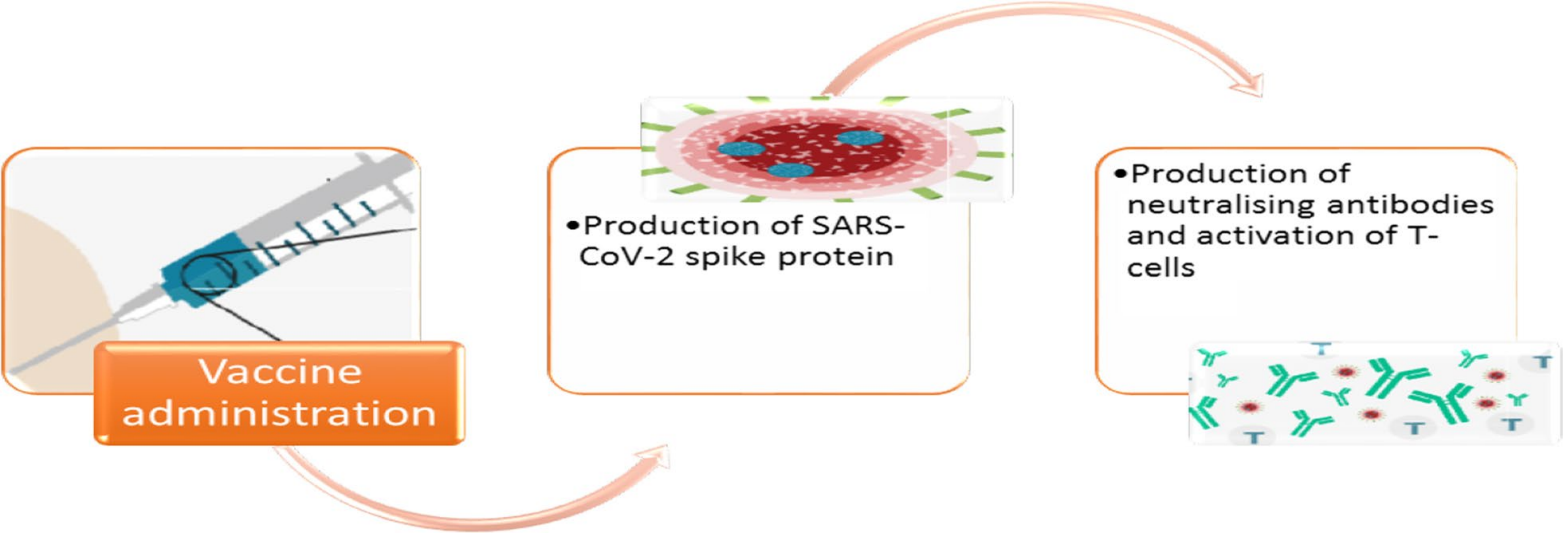
COVID-19 vaccine activation of immune response

The vaccines are expected to stimulate immune responses when administered to confer immunity against COVID-19. Among the vaccines currently approved for use are BNT162b2 (Pfizer-BioNTech), ChAdOx1 (Astrazeneca—Oxford University), Ad26.COV2.S (Johnson and Johnson/Janssen) and m-RNA 1273 vaccine (Moderna—US National Institute of Health (NIH)). BNT162b2 and m-RNA 1273 are mRNA vaccines, using the mRNA technology and lipid nanoparticle (LNP) delivery systems, while ChAdOx1 and Ad26.COV.S are adenovirus vector-based vaccines [5]. The ChAdOx1 vaccine is developed from Ad specie E of a chimpanzee adenovirus-based vector and uses the Cosackie and adenovirus receptor (CAR) [7]. Ad26.COV2.S uses a human Ad26 human-based vector and is made from Ad specie D, which could engage CD46 as its cellular receptor [7]. The mRNA and adenovirus vector-based vaccines induce significant neutralising antibodies and SARS-CoV-2 spike protein antibodies [8, 9] (Fig. 1).

Vaccination against SARS-CoV-2 is an important means of controlling the pandemic, however reports of immune-related adverse effects have raised concerns, although these reactions have been suggested to occur at low prevalence [10]. Among other auto-immune reactions, venous embolism and thrombocytopenia have been observed in some recipients of ChAdOx1-S adenovirus COVID-19 vaccine in several countries, thereby suggesting the occurrence of specific immune-mediated mechanisms and pathogenic syndrome [11, 12]. With the mass vaccination campaign, continuous pharmacovigilance is critical for improved knowledge of vaccine-induced immune thrombotic thrombocytopenia (VITT) syndrome which appears to be rare, but clinically severe. It is important to understand the dynamics of occurrence, the socio-demographic distribution and potential high-risk persons. Adequate and continuous monitoring is essential for early identification and treatment of affected vaccine recipients.

In this review, we evaluated the immune-related clinical and laboratory features associated with BNT162b2 messenger RNA COVID-19 vaccine, Ad26.COV2.S and ChAdOx1 adenoviral vector COVID-19 vaccines. The demographic distribution of pathogenic autoimmune response and time interval between vaccination and onset of symptoms were also assessed. This was to identify; persons at risk of developing an autoimmune response  and markers to enhanced  occurrence of this adverse event.

## Main text

### Study design

The review was performed in accordance with Preferred Reporting Items for Systematic Reviews and Meta-Analysis (PRISMA) guidelines 2009 [13]. Systematic review of eligible articles was conducted from electronic databases of PubMed, Scopus, Web of Science and Google scholar from November 1, 2020 to May 31, 2021.

### Search strategy

With the use of relevant keywords in various combinations, authors conducted an independent search to reduce the risk of potential bias. The keywords were; COVID-19 vaccine, BNT162b2, messenger RNA, Ad26.COV2-S, ChAdOx1, thrombosis, antibodies, SAR-CoV-2, immune reactions and T-cells.

### Study eligibility (inclusion and exclusion criteria)

After a careful assessment of titles and abstracts of studies, only articles that reported studies on immunological and clinical effects of the available COVID-19 vaccines were assessed for eligibility. See Fig. 1. Duplicates where eliminated and full text of papers were independently reviewed by the authors to determine quality of the articles.

Inclusion criteria

(i) Original articles and case reports that reported autoimmune reactions and resulting clinical features of vaccination with BNT162b2.
(ii) Original articles and case reports that reported autoimmune reactions and resulting clinical features of vaccination with ChAdOx1.
(iii) Original articles and case reports that reported autoimmune reactions and resulting clinical features of vaccination with Ad26.CoV2.S.
(iv) Studies written in English language.

Articles were excluded if they were:

(i) Reviews, correspondences, viewpoints and commentaries on COVID-19 vaccines
(ii) Animal studies on COVID-19 vaccine trial
(iii) Clinical trials of COVID-19 vaccines

## Outcome measures

Study outcomes of interest were

(i) Presence of antibodies after vaccination
(ii) Evidence of immunological reactions

### Evaluation of the selected studies and method of risk of bias assessment

Critical evaluation of all included studies was done, and outcomes of interests were noted. Individual studies were assessed for limitations at the study level. Quality of included studies was assessed using the National Heart, Lung and Blood Institute (NHLBI) quality assessment tool [14].

### Data extraction and synthesis

Data were independently extracted by the authors to reduce risk of bias, and these included: study authors of included articles, study location, study design, study size, study outcomes and results. Results were categorised into 4 sections.

### Search results

**Fig. 2:**
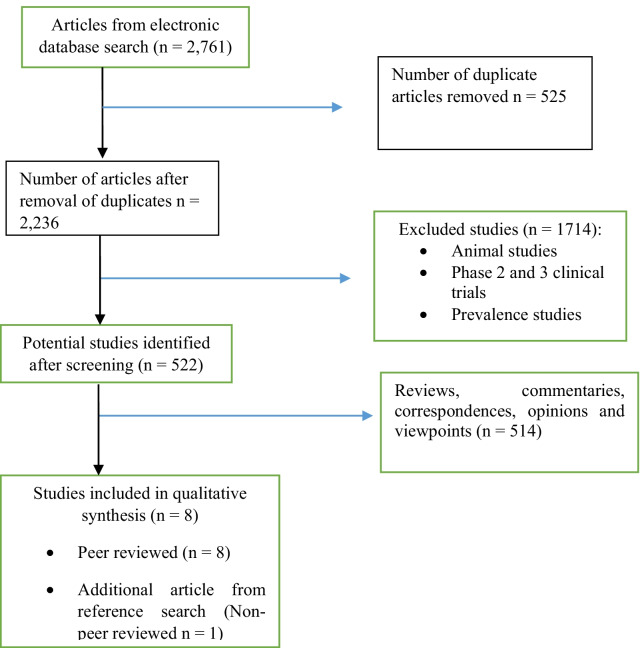
PRISMA flow diagram of study selection

A total of 2,761 resulting titles were screened, from which 522 studies were assessed for eligibility, and 9 studies which met the inclusion criteria were included in the review. Reasons for excluded studies include unavailability of primary data as seen in some correspondences, reviews and commentaries. Other reasons were, low ability of studies to mirror the vaccine effects in the general population, as seen in animal studies and clinical trials . Summary of study selection is shown in the PRISMA flow diagram (Fig. 2).

### Characteristics of included studies

A total of 9 studies discussed the immunogenicity of BNT162b2 mRNA vaccines as well as ChAdOx1 and Ad26.CoV.S adenovirus vector-based vaccines, and all the studies were conducted in 2021. All included studies except 1, were peer-reviewed and the studies comprised 2 case reports, 2 case series and 5 observational studies (see Table 1). The studies were mostly of high quality as assessed by the NHLBI quality assessment.

**Table 1:** Characteristics of included studies

Authors	Study design	Study participants	Sample size	Time from vaccination to onset of symptoms (Days)	Findings	Limitations
Parry et al. 2021 [8]	Observational	Older adults (80–99-years-old)	165	-	Antibody response in a large majority Enhanced T-cells response in ChAdOx1 than in BNT162b2 mRNA recipients	Small sample size Not peer-reviewed (as at the time of this review)
Muller et al. 2021 [9]	Observational	Older persons (≥ 60-years-old)	176	-	After 2 doses of BTN162b2 mRNA vaccine, SARS-CoV-2 spike protein specific IgG antibody was lower in older persons (> 80-years-old) Detectable neutralising antibody titre was absent in many older persons, compared to younger persons	Small sample size
Scully et al. 2021 [15]	Observational		23	6 – 24 days after vaccination with ChAdOx1	Vaccine-induced immune thrombotic thrombocytopeania (VITT) after ChAdOx1 vaccine Presence of Platelet Factor 4 (PF4)	Potential risk of ascertainment bias in associating adverse events with vaccination
Shultz et al. 2021 [17]	Observational	Persons of 32–52-years-old	5	7 – 10 days after vaccination with ChAdOx1	Suggests a possible development of vaccine-induced immune thrombotic thrombocytopenia after ChAdOx1 vaccine Presence of Platelet Factor 4 (PF4)	Potential risk of ascertainment bias
Greinacher et al. 2021 [18]	Observational	Persons from Austria and Germany	11	5 -16 days of vaccination ChAdOx1	Development of immune thrombotic thrombocytopenia (ITT) after vaccination with ChAdOx1 nCov-19 Seen mostly in females Highly elevated D-dimer levels and low fibrinogen	Potential risk of ascertainment bias. Potential under-reporting of cases following passive surveillance
Yocum et al. 2021 [19]	Case report	A 62-year-old female	1	37 days of vaccination with Ad26.COV2-S	Low fibrinogen, low platelets and negative tests for PF4 (37 days after Ad26.COV2-S vaccine administration) Suspected vaccine-induced thrombotic thrombocytopenia	Potential risk of ascertainment bias
Blauenfeldt et al. 2021 [21]	Case report	A 60-year-old woman	1	7 days of vaccination with ChAdOx1	Presence of IgG platelet factor 4. increase in D-dimer, thrombocytopenia and haemorrhage	Potential risk of ascertainment bias
Wolf et al. 2021 [22]	Case series	Women between 22 and 46-years-old	3	4 – 17 days after vaccination with ChAdOx1	Elevated D-dimer, Presence of IgG platelet factor 4, presence of SARS-CoV-2 spike IgG	Potential risk of ascertainment bias
See et al. 2021 [23]	Case series	Women of 18–< 60-years-old	12	6 – 15 days after vaccination with Ad26.COV2.S	Positive results for Heparin–platelet factor 4 antibody, abnormal levels of D-dimer and fibrinogen, thrombocytopenia and haemorrhage	Potential risk of incomplete data from retrospective data collection

### Immunogenicity and clinical features relating to vaccination with BNT162b2 mRNA, Ad26.CoV.S and ChAdOx1 adenovirus vector COVID-19 vaccines

In a comparison of immune responses to BNT162b2 mRNA and ChAdOx1 adenovirus vector COVID-19 vaccines, Parry et al. [8] found a strong and largely similar immune responses in older persons of over 80-years-olds after first dose of either vaccines. The study comprised 165 geriatrics of 80–90-years-old in Britain who had received first dose of the vaccines. The study draws attention to associated equivalence and effectiveness of both vaccines in stimulating antibody responses in the large majority of participants. Meanwhile, enhanced T-cells response was seen in persons who received ChAdOx1 adenovirus vector COVID-19 vaccines than in recipients of BNT162b2 mRNA vaccine. As a critical component of the immune system, T-cells possess capacity to induce autoimmune reactions. The researchers also observed that initial infection with SARS-CoV-2 significantly enhanced immune responses, noting evidence of association between increased humoral and cellular immune response with previous natural infection. In the UK study, presence of key antibodies was reportedly more present in recipients of BNT162b2 mRNA than in recipients of ChAdOx1 adenovirus vector COVID-19 vaccines, respectively. The study is suggestive of the synergistic effects of T-cells and antibodies in immunity against COVID-19, following first doses of BNT162b2 mRNA and ChAdOx1 adenovirus vector COVID-19 vaccines. Small sample size is a potential limitation of the study. This study was also yet to be peer-reviewed as at the time of this review.

Meanwhile, Muller et al. [9] analysed blood samples of 176 participants in 2 cohorts comprising a younger group (< 60-years-old) and an older group (> 80-years-old) for vaccine-induced SARS-CoV-2 spike IgG titres and neutralising antibodies, following 2 doses of BTNI62b2 vaccine. The German study evaluated age-related differences in immune response to the vaccine among participants between January 15, 2021 and February 5, 2021. Although the majority of study participants in both groups produced SARS-CoV-2 spike protein specific IgG antibody, titre was significantly lower in the group of older persons after prime and boost doses of vaccine. This is suggestive of attenuated antibody response to the vaccine in older adults of above 80 years of age, which may be associated with age-related decline in humoral and cellular immunity. In addition, neutralising antibody titre was attained in almost all persons in the younger group unlike in the older group. Some of the older participants above 80-years-old were not sero-protected as detectable neutralising titre was not seen in them. As a result, the researchers suggested a need for possible earlier re-vaccination or higher vaccine dose for improved lasting immunity in older persons. The study also found post-vaccination symptoms (side effects) to be absent in a large majority of the older group as compared to the younger group. However, occurrence of side effects was observed to increase with the second dose of the vaccine. The study provides insight to the age-related limitations regarding immune responses after the recommended 2 doses of BTN162b2 mRNA vaccines. However, its small sample size warrants further studies with a larger population.

Findings from 23 patients in United Kingdom who reported thrombosis and thrombocytopenia between 6 and 24 days post-vaccination showed a major lead to likely occurrence of PF4-dependent syndrome following administration of ChAdOx1 COVID-19 vaccine. Sculley and colleagues [15] observed low levels of fibrinogen with increased levels of D-dimer in all the patients at presentation, and presence of Platelet Factor 4 (PF4) was confirmed in 22 of the patients. The study suggests potential occurrence of a pathologic platelet-activating anti-PF4 antibodies following administration of ChAdOx1 adenovirus vector vaccine and subsequent VITT. A continuum of immunological sequence resulting from anti-platelet Factor 4 (Anti-PF4) antibodies mediates autoimmune reactions and subsequent thrombotic disorders [16]. In the study, no evidence of thrombophilia or causative participants was identified in the study patients who mostly consisted of persons younger than 50-years-old and females. Tests for antiphospholid antibodies, antinuclear antibodies and extractable antigen were negative, while lupus antigen was positive in 5 of the 10 patients tested. Acute atypical thrombosis which majorly involved the cerebral veins, and concurrent thrombocytopenia was observed in the patients. Secondary cerebral heamorrhage was also observed in some patients who had cerebral venous thrombosis, and clinically significant bruising was seen in one patient who did not present with thrombosis. Cerebral venous thrombosis was the prevalent clinical feature among the patients, while other observed features were pulmonary embolism, ischaemic stroke, portal vein thrombosis, deep venous thrombosis and bilateral adrenal haemorrhage. A major limitation of this study is its associated small sample size. The study may also be associated with potential risk of ascertainment bias in association of adverse clinical events with vaccination.

Similarly, Schultz et al. [17] reported occurrence of venous thrombosis and thrombocytopenia in 5 patients in Norway within 7–10 days after first dose of ChAdOx1 nCoV-19 adenoviral vector vaccine. The patients were within 32 and 54 age range, mostly females (4) and had no previous exposure to heparin. The study suggests a possible development of vaccine-induced immune thrombotic thrombocytopenia, although this appears to be a rare occurrence following the very few cases seen among a high number of vaccine recipients. Researchers found high levels of antibodies to platelet factor 4-polyanion complexes, including D-dimer, C-reactive protein, anticardiolipin IgG antibodies—in the patients. High levels of IgG antibodies to PF4-polyanion complexes were detected in all patients, and low fibrinogen levels were also reported in the study. Infection of SARS-CoV-2 in the patients was ruled out by negative tests to SARS-CoV-2 antibodies. Early vaccine response was also observed by the researchers, as anti-spike binding was detected in all the patients. Cerebral venous thrombosis was most common among the clinical features observed in the patient. The study provides insight to antibodies that are associated with ChAdOx1 vaccine, however findings did not include post-second dose immune response.

From the clinical and laboratory characteristics of 11 recipients of ChAdOx1 nCov-19 vaccine who developed thrombosis or thrombocytopenia in Austria and Germany, Greinacher et al. [18] concluded that platelet-activating antibodies PF4 mediates a rare development of immune thrombotic thrombocytopenia (ITT), following vaccination with ChAdOx1 nCov-19. ITT occurred mostly in women, persons of 22–49 age range, and within days 5 and 16 post-vaccination. Unusual venous thrombosis was most observed, particularly cerebral venous thrombosis and splanchnic-vein thrombosis. The disorder which clinically appeared like severe heparin-induced thrombocytopenia was also characterised by highly elevated D-dimer levels and low fibrinogen as observed in the study. Meanwhile, there was no evidence of previous exposure to heparin among study participants. This study suggests a rare association between ChAdOx1 vaccination and ITT. The inclusion of laboratory evaluation enhances the objectivity of findings and represents a major strength of the study.

Although limited data were available to assess immunogenicity of Ad26.COV2-S in the general population, isolated case reports have associated it with development of autoimmune reactions related to ChAdOx1 vaccine [19, 20]. However, Sadoff et al. [7] suggested a possible difference in biological activities of Ad26.COV2-S and ChAdOx1 vaccine, citing differences in receptor sites and vector-base as reasons. Meanwhile, Yacum and Simon [19] presented a report of a 62-year-old female who developed altered mental status, acute kidney injury and possible vaccine-induced thrombotic thrombocytopenia 37 days after receipt of Ad26.COV2-S vaccine. Patient presentation as reported by the authors mimicked heparin-induced thrombotic thrombocytopenia, but there was no history of previous exposure to heparin. Laboratory findings showed low fibrinogen and platelets, with negative tests for PF4. The report provides evidence for closer monitoring of recipients of Ad26.COV2-S vaccine, but is limited by its single case which may not provide substantial representation.

Blauenfeldt et al. [21] described the occurrence of immune-mediated thrombotic response to ChAdOx1 vector-based vaccine in a 60-year-old woman in Denmark. Patient was admitted 7 days post-vaccination, where a significant decrease in platelet counts, and a notable increase in D-dimer were observed in the patient. Platelet Factor 4 reactive antibodies-mediated response consistent with heparin-induced thrombocytopenia was also reported by the authors, and clinically associated with exposure to the vaccine. This is suggestive of immune-mediated response to the vaccine, which was observed to result in thrombocytopenia, haemorrhage and ischaemic stroke in the patient. However, potential risk of ascertainment bias is a major limitation of this report.

Wolf et al. [22] described events of thrombocytopenia and intracranial venous sinus thrombosis in 3 German women, after receipt of ChAdOx1 vaccine. The patients were between 22 and 46-years-old, and symptoms were developed within 4–17 days after vaccination with ChAdOx1 adenovirus vector-based vaccine. Elevated D-dimer, IgG platelet factor 4 and high SARS-CoV-2 spike protein antibodies were observed to be stimulated by the vaccine. The authors also observed thrombocytopenia in the 3 women, meanwhile evidence of thrombophilia was not seen in them. This study generally elicits findings that portray activation of IgG against PF4-heparin complex following ChAdOx1 COVID-19 vaccine in women of child-bearing age who had no significant associated medical history. However, this findings may be limited by lack of a control, for adequate association of clinical event and vaccine administration. Other potential risks associated with observational studies may also pose limitation to findings.

In their report, See et al. [23] described immunological findings from 12 women who received Ad26.COV2-S across 11 states of the United States of America. No history of thrombosis, thrombophilia or prior exposure to heparin was observed in the patients, and only 1 of them had prior exposure to oral contraceptives. However, positive results for Heparin–platelet factor 4 antibody were seen in 11 of the patients, with abnormal levels of D-dimer or fibrinogen in all patients. Thrombocytopenia, intracranial haemorrhage and non-CVST thrombosis clinical features were seen in the patients. Eleven of these women were younger than 50-years-old, and onset of symptoms occurred between 6 and 15 days from vaccination with Ad26.COV2-S, while interval between vaccination and hospitalisation was 10–25 days. The study noted that 11 patients had negative tests for SARS-CoV-2 infection and 1, who reported previous exposure to the infection was not tested. By this, the researchers ruled out the possibility of SARS-COV-2-induced thrombotic thrombocytopenia, as COVID-19 is also associated with high risk of auto-immune inflammatory reactions, including respiratory syndrome and thromboembolism [24, 25]. In this study, time for onset of symptoms differed from the 2-day previously observed time in phase 3 study of the vaccine [26]. A passive surveillance system was used in case identification, and this may have resulted in possible under-reporting of cases. Also, since data were collected retrospectively, potentially relevant information may be incomplete.

## Discussion

Studies showed a possible stimulation of immune response with administration of BNT162b2 mRNA vaccine, ChAdOx1 and Ad26.COV2-S adenovirus vector-based vaccines. Aside SARS-CoV-2 spike protein antibodies, elevated D-dimers, presence of PF4 and low fibrinogen appear to be most commonly seen laboratory features in persons with autoimmune reactions as seen in recipients of these vaccines [15, 17–19, 21–23]. Additionally, haemorrhage, various forms of thrombosis and thrombocytopenia appear to be the commonest clinical features observed in VITT following receipt of ChAdOx1 and Ad26.COV2-S adenovirus vector-based vaccines. Thus, from this review and a previous correspondence [20], authors observed that occurrence of vaccine-induced immune thrombotic thrombocytopenia could relate to adenoviral vector-based vaccines, unlike the messenger RNA vaccine (BNT162b2). However, with larger number of vaccinated people and with longer follow-up, cases of vaccine induced syndromes have been related to the mRNA COVID-19 vaccines [27–29]. In a previous correspondence, Tarawneh and Tarawneh also related BNT162b2 mRNA vaccine to the development of thrombotic thrombocytopenia in a 22-year-old recipient of the vaccine [30]. Similarly, the mRNA 1273 vaccine was also associated to a familial thrombocytopenia flare-up in a 36-year-old female [31].  However, high COVID-19 vaccines effectiveness has been previously reported in the general population [32] and associated risks appear to outweigh potential risks.

Vaccination against SARS-CoV-2 induces humoral and cellular immunity and is critical for the effective control of the pandemic, however pathologic auto-immune syndrome may occur after vaccine administration [15, 17–19, 21–23]. Findings from this study are suggestive of possible higher susceptibility of women to the pathologic immunogenicity that may particularly result from exposure to ChAdOx1 and Ad26.COV2-S adenovirus vector-based vaccines [17–19, 21–23]. This female preponderance has remained unexplained. The syndrome also appears to more likely occur in women of 22 to about 60-years-old, at a time interval of about 4–37 days between vaccination and occurrence of initial symptoms [17–19, 21–23]. Meanwhile, there is need for more data from pharmacovigilance, to ascertain the actual distribution and prevalence of these events. Socio-demographics is also a major influencing factor for beliefs about COVID-19 vaccine [33]. A good knowledge of the socio-demographic distribution of potential VITT is necessary for strategic vaccination to prevent or reduce its occurrence. This will reduce fears of potential vaccine-related side effects, and may likely enhance vaccine acceptability. A previous study reported a COVID-19 vaccine acceptability of 56% which is not sufficient for formation of herd immunity [34].

Although incidence of VITT following pathogenic PF4 syndrome appears to be very low [6, 10, 35], it is observed to present with clinically severe illness requiring early identification and urgent treatment [15, 17–19, 21, 23]. In a communique after its extraordinary meeting, the safety committee of European Medicines Agency (EMA) confirmed the possible association of ChAdOx1 COVID-19 vaccine with rare cases of thrombosis including cerebral venous thrombosis and thrombocytopenia with or without the occurrence of haemorrhage [36]. However, it concluded that vaccine benefits outweigh risks of COVID-19. Therefore, active surveillance is essential while continued vaccination is maintained for pandemic control.

This study has contributed to the body of knowledge for enhanced effectiveness of COVID-19 vaccination, however it is associated with some limitations. Passive surveillance system was used in case identification in the included studies, and this may have resulted in possible under-reporting of cases. Retrospective data collection in some studies particularly the case series may have excluded some potentially relevant information. Inclusion of studies of varying methodologies may have potentially introduced bias. Also, only few studies on BNT16b2 and Ad26.COV2-S were available and included as at the study period, as a result, exaggerated differences in immunogenicity of vaccines may have occurred in the study. Although clinical and laboratory features were used to associate adverse events with vaccination, potential risk of ascertainment bias may not be absent. Additionally, inclusion of only five databases may have excluded some potentially relevant studies.

## Conclusions

BNT162b2 mRA, ChAdOx1 and Ad26.COV2.S were associated with stimulation of immune responses. However, immune thrombotic thrombocytopenia and other PF4 dependent syndrome are likely associated with ChAdOx1 and Ad26.COV2.S adenovirus vector vaccines, mostly occurring in women usually within 4 to 37 days of first dose of vaccine. Enhanced knowledge about vaccine adverse effects and its distribution is crucial for effective vaccination strategies.

## Data Availability

Not applicable.

## Abbreviations

SARS-CoV-2: Severe Acute Respiratory Syndrome Coronavirus 2
COVID-19: Coronaviruses Disease 2019
PF4: Platelet Factor 4
mRNA: Messenger Ribonucleic Acid
VITT: Vaccine Induced Thrombotic Thrombocytopenia
EMA: European Medicines Agency
